# An Unusual Presentation of Metformin-Associated Lactic Acidosis: A Case Report

**DOI:** 10.7759/cureus.78870

**Published:** 2025-02-11

**Authors:** Mathew Zleczewski, Abdalhai Alshoubi

**Affiliations:** 1 Anesthesiology and Critical Care, University of Illinois College of Medicine Peoria, Peoria, USA

**Keywords:** acute kidney injury, hemodialysis, metformin, metformin-associated lactic acidosis, type 2 diabetes mellitus

## Abstract

Metformin-associated lactic acidosis (MALA) is a rare but serious complication of metformin therapy, typically occurring in patients with underlying risk factors, such as renal impairment. While metformin is commonly prescribed for managing type 2 diabetes, its association with lactic acidosis is infrequent. We present the case of a 73-year-old woman with type 2 diabetes and atrial fibrillation (AFib) who developed MALA following acute kidney injury (AKI). The patient presented with symptoms of malaise, dizziness, abdominal pain, nausea, and vomiting, along with signs of severe metabolic acidosis and elevated lactate levels.

Upon further evaluation, she was found to have impaired renal function, which likely reduced the clearance of metformin and lactate, leading to the development of MALA. The patient underwent hemodialysis for four days, which led to a gradual normalization of her acid-base balance and an improvement in renal function. She made a full recovery and was discharged home after two weeks.

Metformin can inhibit mitochondrial function, impairing lactate clearance and increasing the risk of lactic acidosis, particularly in patients with compromised renal function. The clinical presentation of MALA is nonspecific, often mimicking other conditions, such as sepsis or gastrointestinal disorders. Early recognition and management, including hemodialysis, are crucial for improving outcomes. This case highlights the importance of monitoring patients with predisposing factors for MALA and providing timely intervention to prevent severe complications.

## Introduction

Metformin-associated lactic acidosis (MALA) is a rare but serious complication that can arise from metformin therapy in patients with type 2 diabetes mellitus [1]. While metformin is widely prescribed and generally considered safe, its association with lactic acidosis is infrequent. A review involving 11,800 metformin users over approximately two years reported an incidence of only nine cases per 100,000 years of exposure, highlighting the rarity of this condition [2].

This case report details a 73-year-old woman with type 2 diabetes and atrial fibrillation (AFib) who developed MALA, emphasizing the importance of vigilant monitoring in patients with predisposing factors. Understanding the rarity and clinical implications of MALA is essential for optimizing patient outcomes in similar scenarios. Treatment mainly focuses on supportive care to correct acid-base imbalances, discontinue metformin, and treat coexisting conditions.

## Case presentation

A 73-year-old woman with type 2 diabetes and AFib presented to the Emergency Department after experiencing generalized malaise, dizziness, abdominal pain (particularly in the left lower quadrant), poor oral intake, nausea, and vomiting over the past three days. She was diagnosed with type 2 diabetes four years ago and has been taking metformin since. Her medical history also includes hypertension and depression, along with chronic constipation, averaging four bowel movements per month; her last bowel movement occurred two weeks before admission.

Her regular medications included metformin (1000 mg twice daily), amiodarone (200 mg daily), warfarin (5 mg daily), lisinopril (10 mg daily), atorvastatin (40 mg daily), and nortriptyline (10 mg daily). The patient’s height was 1.62 m, weight was 74 kg, resulting in a body mass index of 28 kg/m².

The patient was alert and oriented, with a Glasgow Coma Scale score of 15. Her vital signs indicated a blood pressure of 151/55 mmHg, a pulse of 60 beats per minute, a respiratory rate of 18 breaths per minute, oxygen saturation of 94% on room air, and a body temperature of 37.2°C. On examination, her abdomen was soft but tender, with rebound tenderness noted in the left lower quadrant and diminished bowel sounds. Heart and lung evaluations were normal.

Laboratory tests revealed severe anion gap metabolic acidosis and lactic acidosis, along with impaired renal function (Table 1). However, serum and urine ketones were negative. Urinalysis was unremarkable, and both chest X-ray and abdominal computed tomography angiography showed no abnormalities. She was started on broad-spectrum antibiotics.

**Table 1:** Serum laboratory results on the first and last days of admission WBC: white blood cells; RBC: red blood cells; BUN: blood urea nitrogen; AST: aspartate transferase; ALT: alanine transaminase; INR: international normalized ratio; PT: prothrombin time; PTT: partial thromboplastin time; uL: microliter; g/dL: grams per deciliter; fL: femtoliters; mmol/L: milimoles per liter; mg/dL: milligrams per deciliter; U/L: units per liter

Parameter	Reference range and units	First day of admission	Last day of admission
WBC count	4-10 × 10^3^/uL	13.1	8.1
RBC count	4.3-5.9 × 10^6^/uL	3.75	3.20
Hemoglobin	14-18 g/dL	10.6	9.7
Hematocrit	39-49%	31.9	29.2
Mean corpuscular volume	80-99 fL	84.8	80.4
Red cell distribution width	11.4-14.6%	13.5	16.1
Platelet count	150-400 × 10^3^/uL	212	223
Lymphocytes	16-45%	15.1	21
Neutrophils relative percent	42-75%	77	59
Monocytes	2-12%	6.7	7
Eosinophils	0-5%	0.1	0
Basophils	0-2%	0.3	1
Sodium	135-145 mmol/L	143	139
Potassium	3.5-5.1 mmol/L	4	4.2
Chloride	98-107 mmol/L	95	101
Carbon dioxide	21-32 mmol/L	16	25
Anion gap	<18 mmol/L	32	13
Glucose	74-106 mg/dL	125	147
BUN	7-18 mg/dL	58	41
Creatinine	0.70-1.30 mg/dL	6.77	3.5
Calcium	8.5-10.1 mg/dL	7.6	10.1
Phosphorus	2.3-4.7 mg/dL	8.3	5.4
AST	15-37 U/L	227	16
ALT	16-61 U/L	158	25
Protein, total	6.4-8.2 g/dL	4.6	4.1
Albumin	3.4-5 g/dL	2.7	2.6
Alkaline phosphatase	40-150 U/L	46	52
Bilirubin, total	0.3-1 mg/dL	0.6	2
INR	1	2.6	2.7
PT	9.4-12.5 seconds	13	14
PTT	25.1-36.5 seconds	32	31
Lactic acid	0.5-2 mmol/L	14.1	1.5

Due to the concern of peritonitis, the patient underwent an emergency diagnostic laparoscopy, which showed no significant findings. Blood cultures were negative, and she continued receiving broad-spectrum antibiotics for seven days. As her acute renal failure and lactic acidosis worsened, she began hemodialysis for four days. The patient’s acid-base balance slowly normalized, and kidney function improved. After excluding all causes of high anion gap lactic acidosis, we presumed that our patient had severe MALA in the setting of severe hypovolemic acute renal failure. Two weeks later, the patient made a full recovery and was discharged home.

## Discussion

Metformin is a widely used antidiabetic medication that primarily decreases hepatic glucose production and enhances insulin sensitivity in peripheral tissues, particularly in muscle and adipose tissues. While it effectively lowers blood glucose levels, metformin can also increase plasma lactate levels due to its effects on mitochondrial function. Specifically, metformin inhibits complex I of the mitochondrial respiratory chain, impairing oxidative phosphorylation and resulting in elevated lactate production [3]. When lactate production exceeds its clearance - especially in patients with compromised renal function - the risk of lactic acidosis significantly increases, manifesting as metabolic acidosis with elevated lactate levels. Lactic acidosis is characterized by a pH below 7.35, blood lactate levels greater than 2.0 mmol/L, and a PaCO_2_ under 42 mmHg. Shock is the most common cause of severe lactic acidosis, though metformin-induced lactic acidosis, while rare, can carry a mortality rate as high as 50% [4].

In this case, the patient presented with acute kidney injury (AKI), likely precipitated by factors such as dehydration and poor oral intake, which can significantly impair renal function. This decline in renal function reduced the clearance of both metformin and lactate from the bloodstream, thereby increasing the risk of developing MALA [1]. Elevated lactate levels in such scenarios are often attributed to an imbalance between increased production and decreased clearance [3]. Additionally, the patient’s history of AFib introduces another layer of complexity, as AFib can lead to fluctuations in cardiac output and oxygen delivery. These fluctuations may contribute to tissue hypoxia, promoting anaerobic metabolism and increasing lactate levels. The interplay between her cardiac status and metabolic processes highlights the need for careful monitoring in patients with these comorbidities [5].

Furthermore, the presence of other risk factors, such as chronic constipation and the patient’s history of diabetes, complicates the clinical picture. Chronic constipation may have led to dehydration, further straining her renal function and disrupting acid-base homeostasis. Infections can also provoke metabolic derangements; while the patient did not present with overt signs of illness, subclinical infections could still contribute to elevated lactate levels. Clinicians should be aware that lactic acidosis can occur even with adequate tissue perfusion and oxygenation, emphasizing the need for a comprehensive evaluation [6]. Therefore, when assessing clinical status, it is essential to consider all underlying conditions that can contribute to the development of lactic acidosis, as demonstrated by our case.

Management of MALA primarily focuses on supportive care and resolution of underlying causes. In this case, the patient required hemodialysis to remove accumulated metformin and correct her metabolic disturbances effectively. Ongoing monitoring of lactate levels, renal function, and the anion gap is crucial for guiding treatment decisions and ensuring optimal patient outcomes. It underscores the importance of maintaining a high index of suspicion for MALA in patients with AKI and other risk factors. Educating patients about the signs of lactic acidosis can aid in early detection and intervention. Symptoms of metformin toxicity closely resemble those of sepsis and gastrointestinal disorders, including nausea, vomiting, abdominal pain, and leukocytosis. When gastrointestinal symptoms are present alongside elevated lactate levels, mesenteric ischemia should also be considered as a potential cause [7,8]. The clinical presentation of MALA is often nonspecific, and in severe cases, patients may experience hypotension and respiratory failure, which may require mechanical ventilation [4,9].

A recent study found that continuous venovenous hemofiltration eliminates metformin toxicity less effectively than conventional hemodialysis. Indications for extracorporeal treatment include high lactate levels (>20 mmol/L), low pH (<7.0), shock, inadequate response to standard supportive measures, and decreased level of consciousness [6]. Therefore, continuous hemodialysis may be preferable for patients who are hemodynamically unstable for conventional hemodialysis. The overall mortality rate for MALA was around 50% during the period from 1960 to 2000 but has since decreased to approximately 25% [4]. These trends underscore the importance of early and effective intervention in improving patient outcomes.

## Conclusions

This case highlights the importance of recognizing MALA as a potential complication, especially in patients with renal impairment, AFib, and other risk factors. The patient's AKI likely precipitated her MALA, demonstrating how multifactorial interactions can lead to serious metabolic disturbances. Rapid recognition and appropriate treatment, including hemodialysis, are critical for improving outcomes in affected patients. Proactive management strategies and patient education are essential to mitigate the risks associated with metformin therapy and enhance overall safety in clinical practice.
